# Supplementary material for: Control of complement-induced inflammatory responses to SARS-CoV-2 infection by anti-SARS-CoV-2 antibodies
Source: EMBO J. 2024 Feb 28;43(7):2. doi: 10.1038/s44318-024-00061-0 (PMC10987522; doi:10.1038/s44318-024-00061-0)
Supplement: Supplementary file 9 — Expanded View Figures [file 44318_2024_61_MOESM9_ESM.pdf]

Expanded View Figures

Figure EV1. Spike-dependent opsonization requires C3 deposition for complement opsonization.

(**A**) Particles lacking SARS-CoV-2 Spike glycoprotein and SARS-CoV-2 pseudovirus opsonisation were determined by ELISA (p24 pg/mL) ($n = 3$ donors). (**B**) Deposition of C3b on DC-internalized SARS-CoV-2 after incubation with pre-pandemic NHS, C3-depleted sera and heat-inactivated sera ($n = 3$ donors). (**C**, **D**) Human monocyte-derived DCs were exposed to SARS-CoV-2 isolate (hCoV-19/Italy-WT, 1000 TCID/mL) and complement-opsonized SARS-CoV-2 (hCoV-19/Italy-WT, 1000 TCID/mL) in presence or absence of anti-CD11b and anti-CD11c. LPS stimulation was used as positive control for DC maturation, which was measured after 24 h by flow cytometry. Cumulative flow cytometry data of CD11b and CD11c ($n = 12$ donors). Data show the mean values and error bars are the SEM. Statistical analysis was performed using (**A**) two-way ANOVA with Tukey multiple-comparison test. *$P \le 0.05$, **$P \le 0.01$, ***$P \le 0.001$ ($n = 3$ donors). (**B**) ordinary one-way ANOVA with Dunnett's multiple-comparison test **$P \le 0.01$ ($n = 3$ donors). (**C**, **D**) Two-way ANOVA with Tukey multiple-comparison test. *$P \le 0.05$, ***$P \le 0.001$ ($n = 12$ donors). Source data are available online for this figure.

© The Author(s)

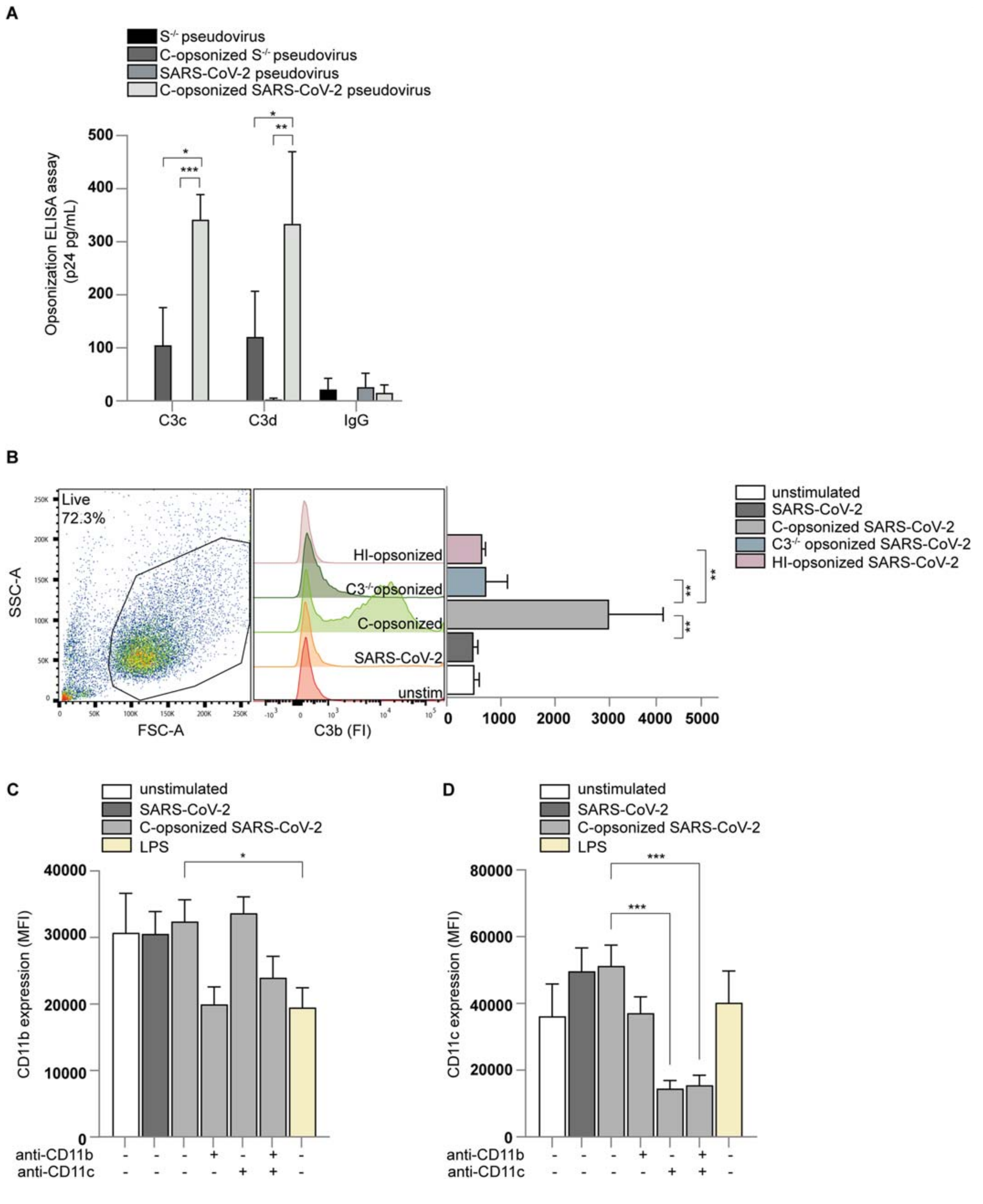
A
S-/- pseudovirus
C-opsonized S-/- pseudovirus
SARS-CoV-2 pseudovirus
C-opsonized SARS-CoV-2 pseudovirus
Opsonization ELISA assay (p24 pg/mL)
C3c
C3d
IgG
B
Live 72.3%
SSC-A
FSC-A
HI-opsonized
C3-/- opsonized
C-opsonized
SARS-CoV-2
unstim
C3b (FI)
unstimulated
SARS-CoV-2
C-opsonized SARS-CoV-2
C3-/- opsonized SARS-CoV-2
HI-opsonized SARS-CoV-2
C
unstimulated
SARS-CoV-2
C-opsonized SARS-CoV-2
LPS
CD11b expression (MFI)
anti-CD11b
anti-CD11c
D
unstimulated
SARS-CoV-2
C-opsonized SARS-CoV-2
LPS
CD11c expression (MFI)
anti-CD11b
anti-CD11c

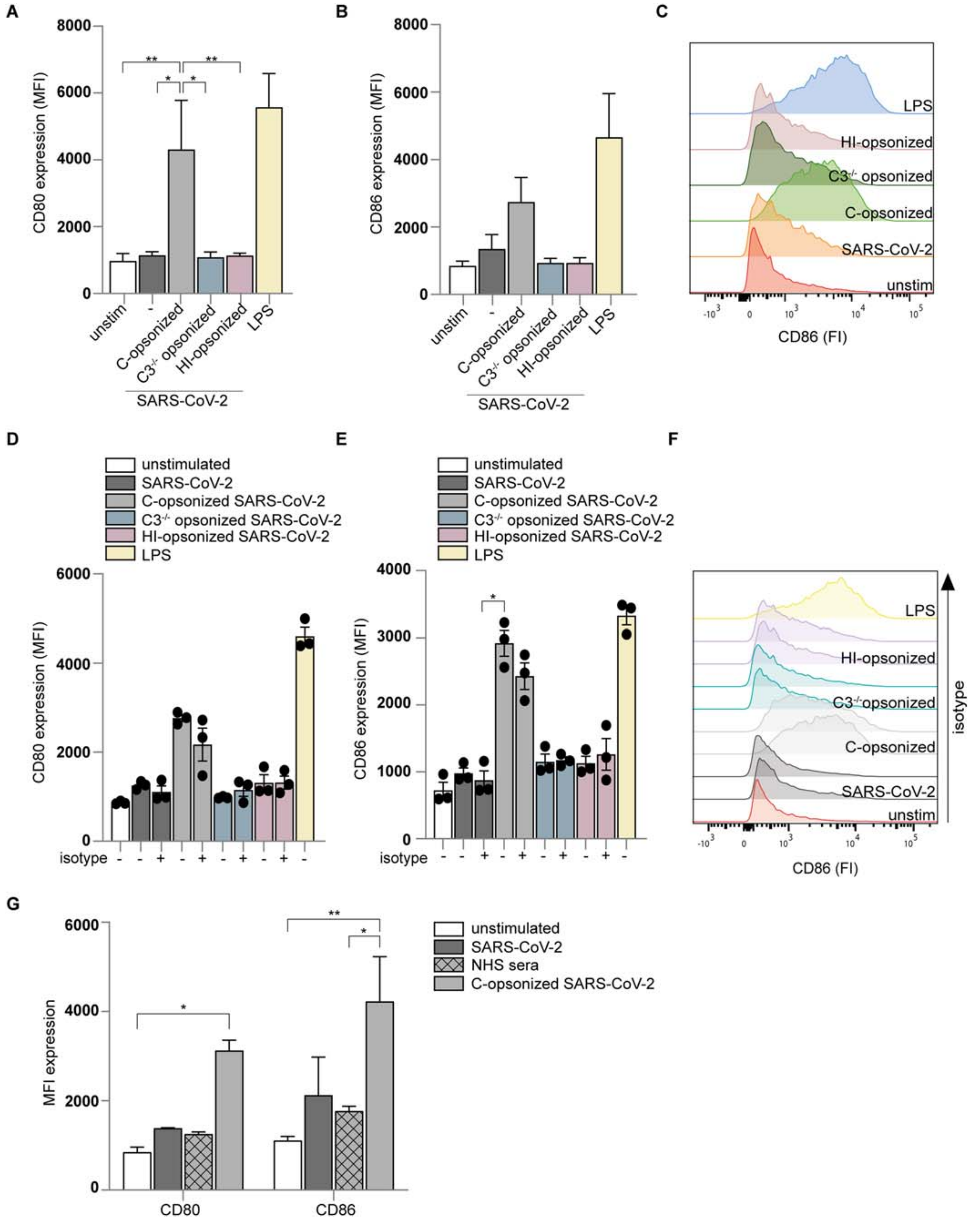
A
CD80 expression (MFI)
8000
6000
4000
2000
0
**
*
*
**
unstim
-
C-opsonized
C3-/- opsonized
HI-opsonized
LPS
SARS-CoV-2
B
CD86 expression (MFI)
8000
6000
4000
2000
0
unstim
-
C-opsonized
C3-/- opsonized
HI-opsonized
LPS
SARS-CoV-2
C
LPS
HI-opsonized
C3-/- opsonized
C-opsonized
SARS-CoV-2
unstim
CD86 (FI)
D
unstimulated
SARS-CoV-2
C-opsonized SARS-CoV-2
C3-/- opsonized SARS-CoV-2
HI-opsonized SARS-CoV-2
LPS
CD80 expression (MFI)
6000
4000
2000
0
isotype
E
unstimulated
SARS-CoV-2
C-opsonized SARS-CoV-2
C3-/- opsonized SARS-CoV-2
HI-opsonized SARS-CoV-2
LPS
CD86 expression (MFI)
4000
3000
2000
1000
0
*
isotype
F
LPS
HI-opsonized
C3-/-opsonized
C-opsonized
SARS-CoV-2
unstim
isotype
CD86 (FI)
G
MFI expression
6000
4000
2000
0
*
**
*
unstimulated
SARS-CoV-2
NHS sera
C-opsonized SARS-CoV-2
CD80
CD86

 © The Author(s)

Figure EV2. C3 deposition is required for DC maturation.

(**A–F**) Human monocyte-derived DCs were exposed to SARS-CoV-2 isolate (hCoV-19/Italy-WT, 1000 TCID/mL), complement-opsonized SARS-CoV-2 (hCoV-19/Italy-WT, 1000 TCID/mL), C3-depleted-opsonized SARS-CoV-2 (hCoV-19/Italy-WT, 1000 TCID/mL) and heat-inactivated-opsonized SARS-CoV-2 (hCoV-19/Italy-WT, 1000 TCID/mL) in absence (**A–C**) or presence (**D–F**) of an isotype. LPS stimulation was used as positive control for DC maturation, which was measured after 24 h by flow cytometry. Cumulative flow cytometry data of CD80 (**A–D**) and CD86 (**B–E**) ($n = 5$ donors). (**C**, **D**) Representative histograms of CD86 expression. (**G**) DCs were exposed to SARS-CoV-2 isolate (hCoV-19/Italy-WT, 1000 TCID/mL), pre-pandemic NHS sera and complement-opsonized SARS-CoV-2 (hCoV-19/Italy-WT, 1000 TCID/mL) and the expression of CD80 and CD86 markers were measured ($n = 4$ donors). Data show the mean values and error bars are the SEM. Statistical analysis was performed using (**A**) ordinary one-way ANOVA with Dunnett's multiple-comparison test. *$P \leq 0.05$, **$P \leq 0.01$ ($n = 5$ donors). (**F**) ordinary one-way ANOVA with Tukey multiple-comparison test. *$P \leq 0.05$ ($n = 5$ donors). (**G**) ordinary one-way ANOVA with Tukey multiple-comparison test. *$P \leq 0.05$, **$P \leq 0.01$ ($n = 4$ donors). Source data are available online for this figure.

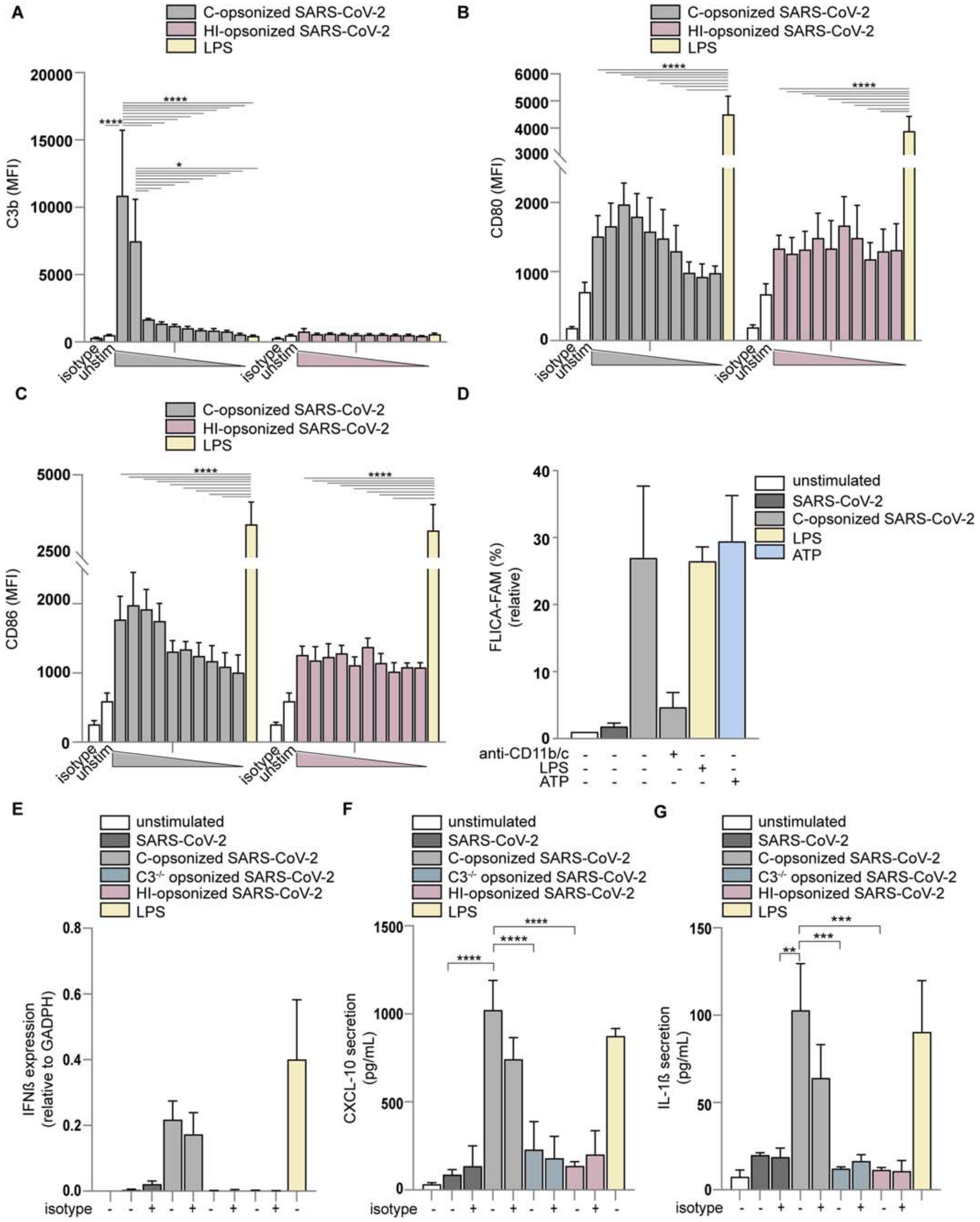
A
C-opsonized SARS-CoV-2
HI-opsonized SARS-CoV-2
LPS
C3b (MFI)
isotype
unstim
B
CD80 (MFI)
C
CD86 (MFI)
D
unstimulated
SARS-CoV-2
C-opsonized SARS-CoV-2
LPS
ATP
FLICA-FAM (%) (relative)
anti-CD11b/c
E
C3-/- opsonized SARS-CoV-2
IFNß expression (relative to GADPH)
F
CXCL-10 secretion (pg/mL)
G
IL-1ß secretion (pg/mL)

 © The Author(s)

Figure EV3. SARS-CoV-2 opsonization is concentration-dependent and requires C3.

(**A**) Mean fluorescence index (MFI) of serum concentration-dependent C3b deposition on DC-internalized complement-opsonized SARS-CoV-2 and heat-inactivated-opsonized SARS-CoV-2 after 24 h ($n = 4$ donors). (**B**, **C**) Mean fluorescence index (MFI) of serum concentration-dependent co-stimulatory markers, CD80 (**B**) and CD86 (**C**) on DC activated cells after exposure to complement-opsonized SARS-CoV-2 and heat-inactivated-opsonized SARS-CoV-2 after 24 h ($n = 4$ donors). LPS stimulation was used as positive control for DC maturation, which was measured after 24 h by flow cytometry. (**D**) Percentages of $FLICA^{+}$ from different stimulated DC ($n = 3$ donors). (**E–G**) Human monocyte-derived DCs were exposed to SARS-CoV-2 isolate (hCoV-19/Italy-WT, 1000 TCID/mL), complement-opsonized SARS-CoV-2 (hCoV-19/Italy-WT, 1000 TCID/mL), C3-depleted-opsonized SARS-CoV-2 (hCoV-19/Italy-WT, 1000 TCID/mL) and heat-inactivated-opsonized SARS-CoV-2 (hCoV-19/Italy-WT, 1000 TCID/mL), as well as LPS (100 ng/mL) in presence or absence of an isotype for 2 h (**E**) and 24 h (**F**, **G**). mRNA levels of IFNβ after 2 h were measured by qPCR ($n = 7$ donors) (**E**). CXCL10 and IL-1β secretion (pg/mL) in the supernatant were measured after 24 h by ELISA ($n = 4$ donors). Data show the mean values and error bars are the SEM. Statistical analysis was performed using (**A–C**), two-way ANOVA with Tukey multiple-comparison test. *$P \leq 0.05$, ****$P \leq 0.0001$ ($n = 4$ donors) (**A–C**). (**E–G**) Two-way ANOVA with Tukey multiple-comparison test. **$P \leq 0.01$, ***$P \leq 0.001$, ****$P \leq 0.0001$ ($n = 7$ donors) (**E**) and ($n = 4$ donors) (**F**, **G**). Source data are available online for this figure.

© The Author(s)

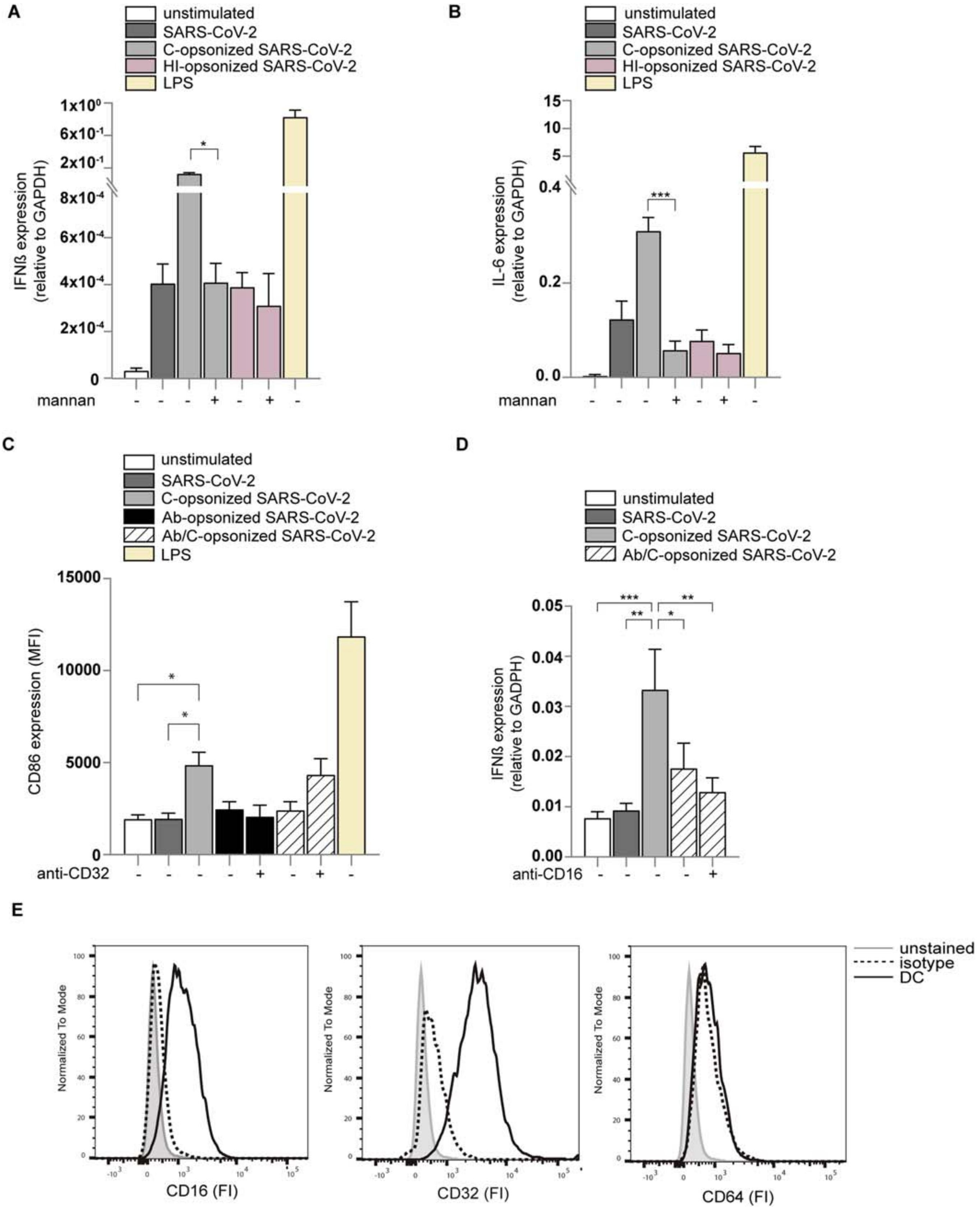
A
unstimulated
SARS-CoV-2
C-opsonized SARS-CoV-2
HI-opsonized SARS-CoV-2
LPS
IFNß expression (relative to GAPDH)
mannan
B
IL-6 expression (relative to GAPDH)
C
Ab-opsonized SARS-CoV-2
Ab/C-opsonized SARS-CoV-2
CD86 expression (MFI)
anti-CD32
D
IFNß expression (relative to GADPH)
anti-CD16
E
unstained
isotype
DC
Normalized To Mode
CD16 (FI)
CD32 (FI)
CD64 (FI)

 © The Author(s)

Figure EV4. Complement-mediated DC activation and antiviral response is dependent on lectin pathway.

(**A**, **B**) NHS and HIS were incubated with mannan (100 μg/mL), prior SARS-CoV-2 opsonization. DCs were exposed to non-, complement-opsonized SARS-CoV-2 and heat-inactivated-opsonized SARS-CoV-2 in presence or absence of mannan, and mRNA levels of IFNβ after 2 h ($n = 3$ donors) (**A**) and IL-6 after 6 h ($n = 3$ donors) (**B**) were determined by qPCR. (**C**) Human monocyte-derived DCs were exposed to SARS-CoV-2 isolate (hCoV-19/Italy-WT, 1000 TCID/mL), to complement-opsonized SARS-CoV-2 (hCoV-19/Italy-WT, 1000 TCID/mL), to antibody-opsonized SARS-CoV-2 (hCoV-19/Italy-WT, 1000 TCID/mL) and to antibody/complement-opsonized SARS-CoV-2 (hCoV-19/Italy-WT, 1000 TCID/mL) in presence or absence of anti-CD32 for 24 h. LPS stimulation was used as positive control for DC maturation, which was measured after 24 h by flow cytometry. Cumulative flow cytometry data of CD86 ($n = 12$ donors). (**D**) Human monocyte-derived DCs were exposed to SARS-CoV-2 isolate (hCoV-19/Italy-WT, 1000 TCID/mL), to complement-opsonized SARS-CoV-2 (hCoV-19/Italy-WT, 1000 TCID/mL) and to antibody/complement- opsonized SARS-CoV-2 (hCoV-19/Italy-WT, 1000 TCID/mL) in presence or absence of anti-CD16 for 2 h, and mRNA levels of IFNβ ($n = 6$ donors) were determined by qPCR. (**E**) DCs were stained with antibodies against the surface markers CD16, CD32 and CD64 and analyzed by flow cytometry. Representative histograms for an experiment repeated more than three times with similar results ($n = 3$ donors). Data show the mean values and error bars are the SEM. Statistical analysis was performed using (**A**, **B**) two-way ANOVA with Tukey multiple-comparison test. *$P \leq 0.05$, ***$P \leq 0.001$ ($n = 3$ donors). (**C**) ordinary one-way ANOVA with Tukey multiple-comparison test. *$P \leq 0.05$ ($n = 6$ donors). (**D**) Two-way ANOVA with Tukey's multiple-comparison test. *$P \leq 0.05$, **$P \leq 0.01$, ***$P \leq 0.001$ ($n = 6$ donors). Source data are available online for this figure.

© The Author(s)

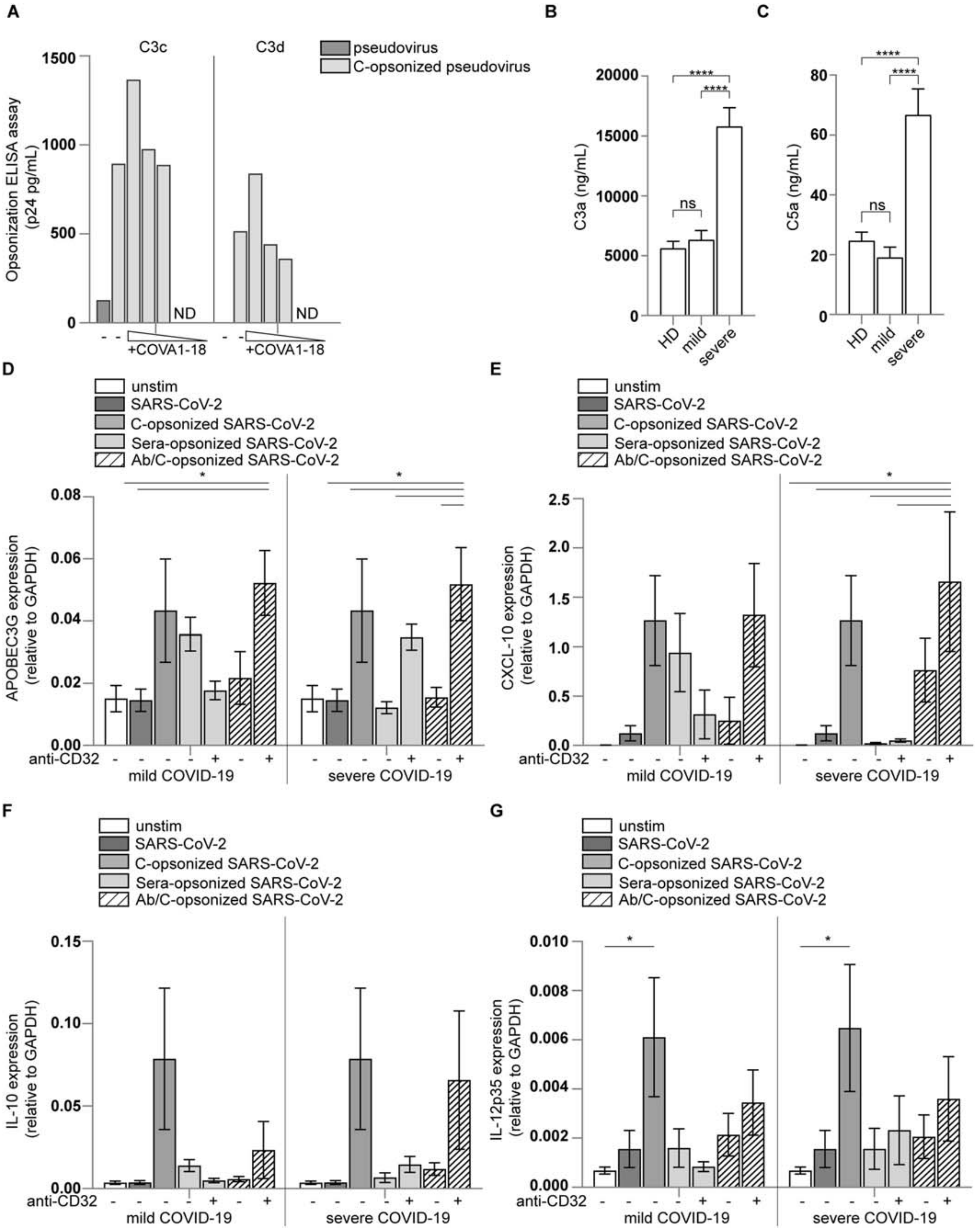
A
C3c
C3d
pseudovirus
C-opsonized pseudovirus
Opsonization ELISA assay (p24 pg/mL)
1500
1000
500
0
ND
ND
+COVA1-18
+COVA1-18
B
C3a (ng/mL)
20000
15000
10000
5000
0
****
****
ns
HD
mild
severe
C
C5a (ng/mL)
80
60
40
20
0
****
****
ns
HD
mild
severe
D
unstim
SARS-CoV-2
C-opsonized SARS-CoV-2
Sera-opsonized SARS-CoV-2
Ab/C-opsonized SARS-CoV-2
APOBEC3G expression (relative to GAPDH)
0.08
0.06
0.04
0.02
0.00
anti-CD32
mild COVID-19
severe COVID-19
E
CXCL-10 expression (relative to GAPDH)
2.5
2.0
1.5
1.0
0.5
0.0
anti-CD32
mild COVID-19
severe COVID-19
F
IL-10 expression (relative to GAPDH)
0.15
0.10
0.05
0.00
anti-CD32
mild COVID-19
severe COVID-19
G
IL-12p35 expression (relative to GAPDH)
0.010
0.008
0.006
0.004
0.002
0.000
anti-CD32
mild COVID-19
severe COVID-19

 © The Author(s)

Figure EV5. Increased complement activation is a distinctive feature of severe COVID-19 patients.

(**A**) NHS concentration-dependent SARS-CoV-2 pseudovirus opsonization by C3c and C3d were determined by (**A**) ELISA (p24 pg/mL) ($n = 2$ donors) (**B**, **C**) C3a and C5a level were determined in healthy donors, mild and severe COVID-19 patients ($n = 7$ donors per group). Plasma samples were harvested and C3a (ng/mL) and C5a (ng/mL) levels were analyzed using a BD Biosciences OptEIA Human C3a and C5a ELISA kit. (**D–G**) Human monocyte-derived DCs were exposed to SARS-CoV-2 isolate (hCoV-19/Italy-WT, 1000 TCID/mL), to complement-opsonized SARS-CoV-2 (hCoV-19/Italy-WT, 1000 TCID/mL), COVID-19 patient serum (mild or severe) and antibody/complement-opsonized SARS-CoV-2 (hCoV-19/Italy-WT, 1000 TCID/mL) in presence or absence of anti-CD32 for 6 h. mRNA levels of APOBEC3G (**D**) CXCL10 (**E**), IL-10 (**F**) and IL-12p35 (**G**) after 6 h were determined by qPCR ($n = 8$ donors). Data show the mean values and error bars are the SEM. Statistical analysis was performed using (**B**, **C**) ordinary one-way ANOVA with Tukey multiple-comparison test. ****$P \leq 0.0001$ ($n = 7$ donors). (**D**, **E**, **G**) Two-way ANOVA with Tukey's multiple-comparison test. *$P \leq 0.05$ ($n = 8$ donors). Source data are available online for this figure.

© The Author(s)
